# Erylusamides: Novel Atypical Glycolipids from *Erylus cf*. *deficiens*

**DOI:** 10.3390/md14100179

**Published:** 2016-10-11

**Authors:** Helena Gaspar, Adele Cutignano, Laura Grauso, Nuno Neng, Vasco Cachatra, Angelo Fontana, Joana Xavier, Marta Cerejo, Helena Vieira, Susana Santos

**Affiliations:** 1 Centro de Química e Bioquímica (CQB), Departamento de Química e Bioquímica, Faculdade de Ciências, Universidade de Lisboa, Campo Grande, Lisboa 1749-016, Portugal; ndneng@ciencias.ulisboa.pt (N.N.); vasco_cachatra@hotmail.com (V.C.); 2 MARE-Centro de Ciências do Mar e do Ambiente, Faculdade de Ciências, Universidade de Lisboa, Campo Grande, Lisboa 1749-016, Portugal; 3 CNR-Istituto di Chimica Biomolecolare, Bio-Organic Chemistry Unit, via Campi Flegrei 34, Pozzuoli (NA) 80078, Italy; acutignano@icb.cnr.it (A.C.); laura.grauso@icb.cnr.it (L.G.); afontana@icb.cnr.it (A.F.); 4 Department of Biology and Centre for Geobiology, University of Bergen, P.O. Box 7803, Bergen N-5020, Norway; joana.xavier@bio.uib.no; 5 Research & Innovation Accelerator, Faculdade de Ciências e Tecnologia, Universidade Nova de Lisboa, Campus de Caparica, Caparica 2829-516, Portugal; m.cerejo@fct.unl.pt; 6 BioISI, Instituto de Biociências e Ciências Integrativas, Faculdade de Ciências, Universidade de Lisboa, Campo Grande, Lisboa 1749-016, Portugal; hmvieira@ciencias.ulisboa.pt

**Keywords:** *Erylus*, indoleamine 2,3 dioxygenase, glycolipids, marine natural products, sponges, anti-cancer, erylusamides

## Abstract

Among marine organisms, sponges are the richest sources of pharmacologically-active compounds. Stemming from a previous lead discovery program that gathered a comprehensive library of organic extracts of marine sponges from the off-shore region of Portugal, crude extracts of *Erylus cf*. *deficiens* collected in the Gorringe Bank (Atlantic Ocean) were tested in the innovative high throughput screening (HTS) assay for inhibitors of indoleamine 2,3-dioxygenase (IDO) and showed activity. Bioassay guided fractionation of the dichloromethane extract led to the isolation of four new glycolipids, named erylusamide **A**–**D**. The structures of the isolated compounds were established by 1D and 2D nuclear magnetic resonance (NMR) spectroscopy, high-resolution electrospray ionization mass spectrometry (HR-ESI-MS) and chemical derivatization. The metabolites shared a pentasaccharide moiety constituted by unusual highly acetylated d-glucose moieties as well as d-xylose and d-galactose. The aglycones were unprecedented long chain dihydroxyketo amides. Erylusamides **A**, **B** and **D** differ in the length of the hydrocarbon chain, while erylusamide **C** is a structural isomer of erylusamide **B**.

## 1. Introduction

The secondary metabolites found in marine invertebrates represent a rich source of novel chemical diversity for lead compounds, with sponges being the most prolific source of new molecules. Between these structurally unique metabolites, glycolipids play an important role. Glycolipids belong to the broad class of glycoconjugates and are characterized by having one or more monosaccharide residues linked by a glycosidic bond to a hydrophobic moiety, such as an acylglycerol, a sphingoid, or a prenyl phosphate [1]. Glycolipids, including glycosphingolipids and gangliosides, are widely found in marine invertebrates, especially in echinoderms (sea stars, sea cucumbers) and sponges, and show a large variety of biological activities such as antitumor, immunomodulatory and nitric oxide release-inhibiting activities [2].

Sponges of the genus *Erylus* Gray, 1867 (Tetractinellida, Geodiidae) were reported to produce uncommon phospholipid methyl branched fatty and unusual glycolipids, some of which have interesting pharmacological activities, such as anticancer and interleukin-6 (IL-6) receptor antagonists (Table 1). The same type of glycolipids found in *Pachymatisma johnstonias*, a species that belongs to the same family of *Erylus*, showed inhibitory activity of bacterial type III secretion [3].

Indoleamine 2,3-dioxygenase (IDO1), formerly known as IDO before the discovery of a second isoform, is the first and rate-limiting enzyme in the oxidative degradation of the essential amino acid tryptophan through the kynurenine pathway and plays a role in the control of infection and in evasion of T-cell-mediated immune rejection [10]. It is believed that IDO1 inhibits the proliferation and differentiation of T cells, which are sensitive to the degradation of tryptophan and accumulation of its catabolites. IDO1 is overexpressed in a variety of tumor cell types and acts against the T-cell attack, thus facilitating the growth and survival of malignant cells [11]. For these reasons, IDO1 has emerged as a key target in cancer immunotherapy. Several inhibitors have been synthesized and proved to be efficient, alone or in combination with other therapeutics. However, by 2014, the pipeline of IDO inhibitors comprised only four drug candidates: indoximod, epacadostat, NLG919 and an IDO derived peptide [12]. Indoximod (d-1-methyl-tryptophan) is being tested in combination with other drugs in several phase I and II clinical trials. Epacadostat (INCB024360), an hydroxyamidine that targets and binds to IDO1 is now in several phase I and II clinical trials [13]. NLG919 is an imidazoleisoindole derivative undergoing phase I clinical trials in the treatment of recurrent advanced solid tumors alone or in combination with other drugs. After the human IDO1 structure was determined by X-ray crystallography in 2006, several synthetic inhibitors were developed based on the structure of the active-site [14]; however, to the best of our knowledge, no comprehensive screening of compounds (or extracts) from marine origin was ever undertaken.

**Figure 1:**
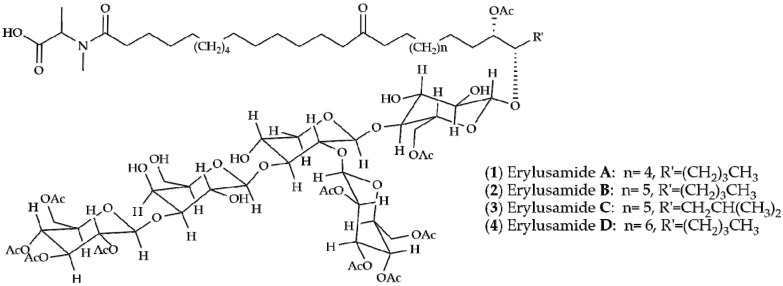
Structures of erylusamides **A**–**D**.

With that background in view, in a previous project, we have undertaken a comprehensive screening of crude extracts of sponges from the Portuguese coast using the Blockade application of GPS D^2^ High Throughput Screening (HTS) system that uses the human version of indoleamine 2,3-dioxygenase 1 (IDO1) as therapeutic target [15]. This paper describes the isolation and structure determination of four new glycolipids, named erylusamides **A**–**D**, compounds **1**–**4** (Figure 1), found in the IDO’s inhibitor organic extract of *Erylus cf*. *deficiens* Topsent, 1927.

## 2. Results and Discussion

Within the scope of a previous drug discovery campaign, a comprehensive library of 185 organic extracts of sponge specimens collected in several off-shore Portuguese locations (Berlengas, Azores and Gorringe bank) was constructed. The extracts were screened as modulators of proteins involved in cancer and neurodegenerative diseases using the Global Platform Screening for Drug Discovery (GPS D2) technology developed by the Portuguese biotech company BIOALVO (Lisbon, Portugal), which uses modified *Saccharomyces cerevisiae* strains designed to express specific targets involved in diseases with a tremendous social and economic burden. BIOALVO’s BLOCKADE application, which targets compounds able to inhibit the enzyme indoleamine 2,3 dioxygenase (IDO-1), was selected to first test the extracts. Extracts were considered positive if they inhibited the growth of BLOCKADE yeast >60% [15]. In the BLOCKADE screening, the dichloromethane extract of the marine sponge *Erylus cf*. *deficiens* collected in the Gorringe Bank (Atlantic Ocean) tested positive at a concentration of 0.125 mg/mL. The activity of this extract was confirmed using an additional assay with African green monkey kidney fibroblast COS7 cells transfected with IDO, revealing an IDO inhibitory activity of 80%. The organic extract was further separated by flash chromatography on C18 reverse phase silica gel (RP-18) into eleven fractions, one of which (fraction 2) conserved the activity of the original extract, inhibiting kynurenine production by 80% at the same concentration.

^1^H nuclear magnetic resonance (NMR) spectrum of fraction 2 (150 mg) revealed complex signals belonging to sugar components between δ 6.4 and 3.5 ppm, together with aliphatic resonances, due to a lipid moiety in the upfield region of the spectrum, thus suggesting the occurrence of a series of glycoconjugates. Hence, as a first step in the structure elucidation of the bioactive components, a methanolysis reaction was performed on an aliquot of the mixture to liberate the aglycone from the monosaccharide pool. Methyl glycosides were converted into the corresponding trimethylsilyl (TMS) derivatives [16] and analysed by GC-MS in comparison with authentic standards. According to retention time and characteristic MS fragmentation patterns, monosaccharide units were identified as d-xylose, d-glucose and d-galactose. On the other hand, aglycones showed IR bands at 3349, 1740, 1701 and 1636 cm^−1^, suggesting the presence of hydroxyl, ester, ketone and amide functionalities, which were confirmed by NMR data. Separation of individual components was achieved by RP-HPLC on a phenyl-hexyl column (Phenomenex) affording **1**–**4** (Figure 1), as pure compounds, here named erylusamides **A**–**D**. High-resolution electrospray ionization mass spectrometry (HR-ESI-MS) analysis in negative ionization polarity revealed that compounds **1**–**4** constituted a series of homologous compounds displaying molecular mass ions at *m*/*z* 1782.8345, 1796.8515, 1796.8434 and 180.8644.

**Figure 2:**
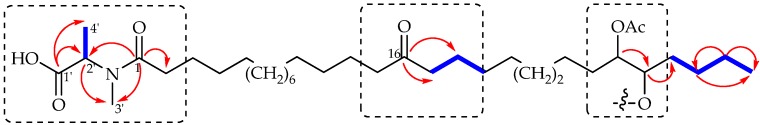
Key HMBC (red ashes) and HSQC-TOCSY (blue lines) correlations establishing the structure of the aglycone moiety.

Erylusamide A (**1**) gave a molecular ion [M − H]^−^ at *m*/*z* 1782.8345, which accounts for the molecular formula C_83_H_133_NO_40_ requiring 18 degrees of formal unsaturation. 1D and 2D-NMR data (Table 2 and Table 3) revealed diagnostic signals of an oligosaccharide moiety composed of five sugar residues, and of a polyketide aglycone displaying three carbonyl signals at δ 210.5, 174.9 and 173.2 ppm in the ^13^C NMR spectrum. Several different spin systems were identified in the aglycone moiety through COSY and HSQC-TOCSY connectivities, and joined by HMBC correlations (Figure 2). In particular, one terminal end of the aglycone polyketide chain was assigned to a *N*-methylalanine substructure. In fact, a deshielded signal at δ 5.75 (H-2′, q) was coupled in the COSY spectra with a methyl doublet at δ 1.54 (H_3_-4′), as well as in the HMBC spectra, and showed correlations with a carboxyl function at δ 174.9 ppm (C-1′) and a methyl carbon on a nitrogen atom at δ 31.5 ppm (C-3′). In turn, the corresponding proton of this later signal was coupled to the carbonyl group at δ 173.2 ppm. The *N*-methylalanine moiety displayed two sets of signals (ratio 3:1) in ^1^H NMR spectrum of **1**, consistent with a *syn*/*anti* rotamer equilibrium typically observed with tertiary amides [17], the major conformer being the *syn* one as deducted from the NOESY correlation H-2 and H-3′. Indeed, this phenomenon was also observed for structurally related pachymoside A, a glycolipid isolated from the marine sponge *Pachymatisma johnstonia* [3].

**Figure 3:**
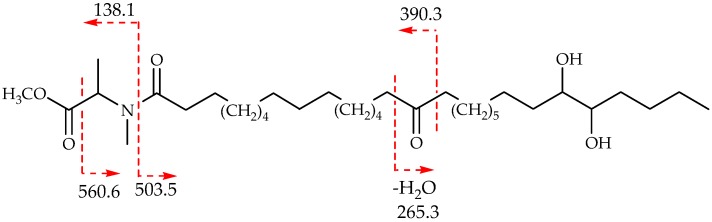
ESI-MS/MS analysis of the aglycone methyl ester **5** at *m*/*z* 620.5 [M + Na]^+^.

The presence of two vicinal oxymethine groups constituting an isolated stereocluster was the most striking feature of the aglycone moiety. In the HSQC spectrum, the crosspeaks at δ 80.7/δ 4.03 and δ 74.9/δ 5.53 suggested the presence of two non-equivalent secondary *O*-substituted alcohols. An HMBC cross-peak was observed between the proton at δ 4.03 and the carbon at δ 74.8 ppm. However, no COSY correlation was observed between the two oxymethine signals suggesting that the dihedral angle between the two protons should be around 90° [18]. These data were consistent with a vicinal diol, with one hydroxyl group acylated and the other one linked to a sugar moiety [19,20]. Furthermore, a connection could be assigned between this diol moiety and terminal *n*-butyl, as depicted from the H2B crosspeak between C-26 (δ 80.7) and the proton at δ 1.79 ppm (H-27), as well as HSQC-TOCSY long range correlations 30.9 → 32.0 → 22.8 → 14.7 → 0.84. The remaining deshielded signal at δ 210.5 corresponded to an aliphatic symmetrical ketone, as deduced from the HMBC correlation with two separated CH_2_ signals at δ 2.42 (4H) and 1.64 (4H) ppm. Compound **1** was methanolysed to liberate the aglycone methyl ester (compound **5**, Figure 3), which was further converted in the corresponding acetonide, and their MS and NMR spectra (see Section 3.4 and Section 3.7) analysed and compared with those of compound **1**. Compound **5** showed a molecular adduct ion [M + Na]^+^ at *m*/*z* 620.5 (Figure 3) compatible with the methyl ester of the deacetylated free aglycone. Comparison of this result with the ones obtained from MS analysis of compound **1** confirmed the presence of an acetyl group on the aglycone moiety: the MS/MS data on molecular ion [M − H]^−^ at *m*/*z* 1782.8 of compound **1** showed a fragment ion at *m*/*z* 624.5, due to the loss of the oligosaccharide portion, compatible with an monoacetylated aglycone moiety. Additionally, a detailed analysis of tandem mass spectrometry (ESI^+^-MS/MS) data (Figure 3) obtained on the aglycone methyl ester **5**, at *m*/*z* 620.5, suggested the location of the carbonyl function at C-16 in the aliphatic chain. In fact, product ion spectra contained diagnostic ions at *m*/*z* 265.3 [C_15_H_30_O_2_ + Na]^+^ and 390.3 formally arising from α-cleavage of the carbonyl group. Furthermore, a fragment ion at *m*/*z* 138.1 confirmed the presence of the *N*-methylalanine moiety.

Finally, analysis of NMR spectra of the acetonide **6** (see Section 3.7) confirmed the occurrence and relative stereochemistry of the 1,2 diol system: the two oxymethine protons at δ 3.72 and 3.74 were coupled by H2BC to the downfield shifted carbons at δ 81.4 and 1.5, respectively, as well as by HMBC with the oxygenated carbon signal at δ 107.8, bearing, in turn, the two acetonide methyl groups at δ 1.50). According to the carbon chemical shifts of these methyl groups of **6**, overlapping at 27.0 ppm, the relative stereochemistry of the 1,2-diol was proposed as *threo*. [21,22,23].

The aglycone part as described above accounted for four out of the 18 formal unsaturations predicted by the molecular formula of **1**. Thus, the remaining 14 double bond equivalents were attributable to the glucosidic portion. The analysis of the ^1^H, ^13^C and HSQC spectra revealed five anomeric carbons, accounting for five sugar rings. The remaining formal unsaturations were assigned to nine acetate residues, which fulfilled the observed [M − H]^−^ ion peak at *m/z* 1782.8345.

Hydrolysis of compound **1** showed that d-xylose, d-galactose and d-glucose were the only monomers present with a ration 1:1:3. The sequence of these sugar residues was determined by extensive NMR study, especially based on 2D techniques (COSY-45, HSQC, HSQC–TOCSY, H2BC, HMBC and NOESY) (Table 2).

**Figure 4:**
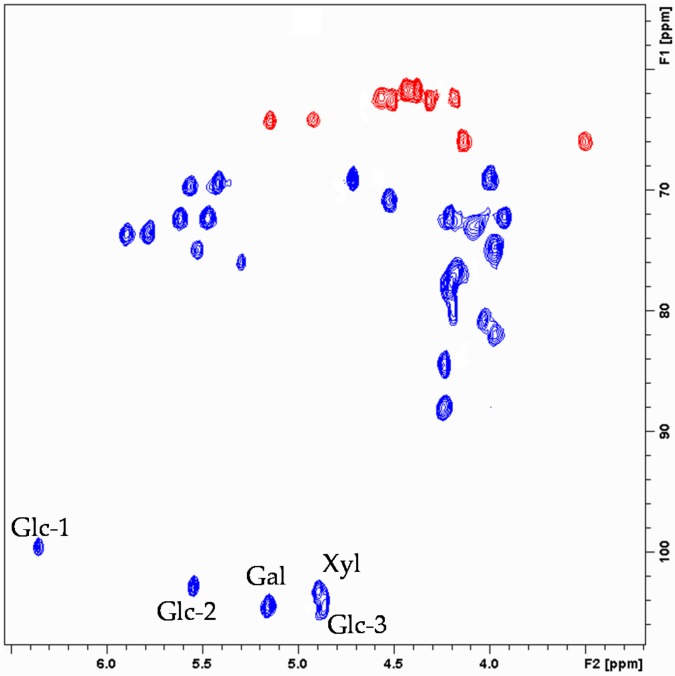
Expansion of HSQC spectrum of erylusamide **A** (**1**) showing the anomeric carbon, the oxymethines and oxymethylenes correlations.

The five anomeric carbons and their attached protons were unequivocally identified at δ^13^ C/^1^H: 104.6/4.86 (d, *J* = 7.9 Hz); 104.4/5.16 (d, *J* = 7.5 Hz); 103.2/4.90 (d, *J* = 7.3 Hz); 102.8/5.55 (d, *J* = 8.0 Hz) and 99.6/6.37 (d, *J* = 7.9 Hz) (Figure 4). The anomeric configurations were assigned as β from the magnitude of the ^3^*J*_1,2_, values, all within the 7–9 Hz interval, typical of diaxial proton coupling [24]. Moreover, the ^13^C NMR shifts of the anomeric carbons, approximatively 100 ppm, also indicate that the corresponding sugars are connected through β-glycosidic bonds [25,26].

**Figure 5:**
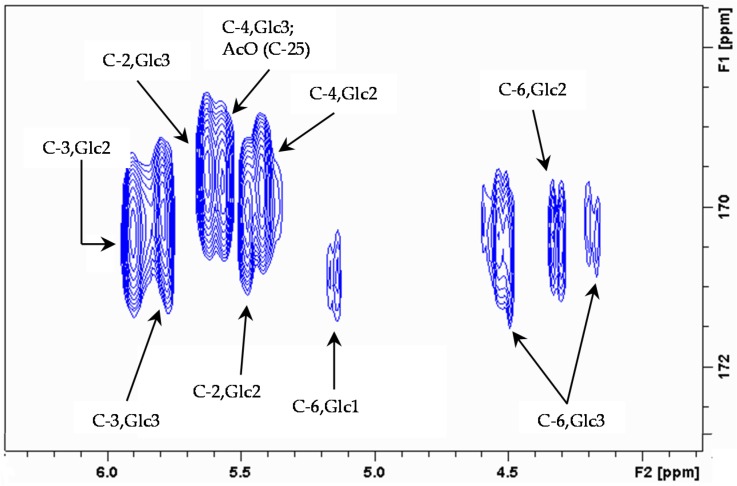
Expansion of HMBC spectrum showing correlations to acetate carbonyls in sugar moiety.

Six of the oxymethines (δH 5.90, 5.78, 5.62, 5.58, 5.48 and 5.42) and three of the oxymethylenes (δH 4.51/4.32, 4.57/4.19, 5.16/4.92) had proton resonating at 1–2 ppm downfield with respect to free hydroxyl groups [19], which indicated the sites of acetylation (Figure 4). The position of acetyl groups was ascertained by HMBC correlations between the acetyl carbonyls and the corresponding oxymethine protons (Figure 5).

The long-range HMBC correlation between C-26 (δ 80.7 ppm) and the β-anomeric proton at δ 4.86 disclosed the linkage between the aglycone portion and the first unit of the pentasaccharide chain, which was assigned to a monoacetylated glucose residue (Glc1). In fact, starting from the anomeric proton TOCSY experiments allowed to delineate the entire spin system while relative configuration was achieved by analysis of NOESY data and *J* couplings. Furthermore, H-6 methylene resulted deshielded thus suggesting the first acetylation site. The MS/MS fragment at *m*/*z* 828.5 [aglyconeGlc1 − H]^−^) from the ion [M − H]^−^ at *m*/*z* 1782.8 is compatible with a monoacetylated glucose.

**Figure 6:**
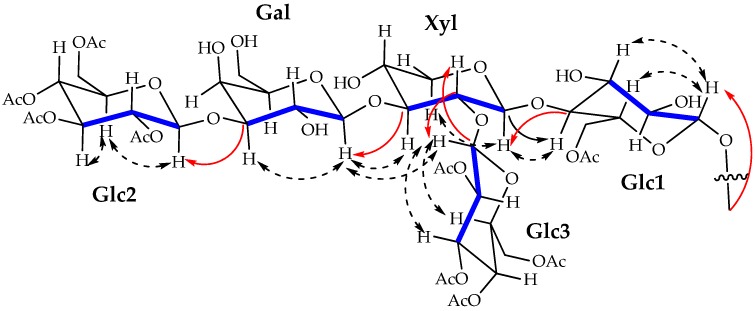
Key HMBC (red ashes), NOESY (dashed ashes) and HSQC-TOCSY (blue lines) correlations establishing the structure of the pentasaccharide moiety.

The HMBC cross peak between C-4 Glc1 and the anomeric proton at 4.90 identified the glycosidic bond between this glucose and the xylose residue, confirmed by the correlation between C-1 Xyl and H-4 Glc1. Xylose showed another glycosidic bond with another glucose residue, which was depicted from cross peaks C-2 Xyl/H-1 Glc3 and C-1 Glc3/H-2 Xyl. A third β-glycosidic bond between xylose and a galactose residue was apparent from the long range correlation C-3 Xyl/ H-1 Gal and the NOESY correlation H-3 Xyl/H-1Gal. Finally, the galactose residue was connected to another glucose unit through the cross peak between C-3 Gal and H-1 Glc2 (Figure 6).

HR-ESI-MS of erylusamides B (**2**) and C (**3**) showed [M − H]^−^ ions at *m*/*z* 1796.8515 and 1796.8434 respectively, consistent with the empirical formula C_84_H_135_NO_40_, suggesting an isomeric relationship, which was reflected in a different behaviour of the two metabolites in HPLC analysis. A careful comparison of NMR spectra indicated structures with aglycones closely related to erylusamide A, which differed for an extra methylene, also confirmed by the peak at *m*/*z* 638.5 [aglycone − H]^−^ (C_37_H_68_O_7_N) in both ESI^−^-MS/MS analyses.

^1^H and ^13^C NMR spectra of compound **2** were almost superimposable with those of **1**, suggesting that the additional methylene should be positioned within the long hydrocarbon chain. Furthermore, NMR data showed that the only difference between the isomeric compounds **2** and **3** was at one chain end of the aglycone moiety, where an isobutyl group in **3** replaced the terminal *n*-butyl residue of **2**. In fact, the ^1^H NMR spectrum of **3** showed the presence of a doublet at δ 0.85 ppm (6 H, *J* = 6.0 Hz) and a multiplet signal at δ 1.51 assigned, respectively, to the methyl and methine protons of the isobutyl moiety. The signal at δ 1.16 was attributed to the remaining methylene group. The two equivalent methyl carbons of the isobutyl moiety were observed at δ 22.8 ppm, while the methine carbon and the methylene appeared, respectively, at δ 28.2 and δ 39.1 ppm.

Erylusamide D (**4**) had a molecular formula of C_85_H_136_O_40_N as revealed by HR-MS-ESI [M − H]^−^ molecular ion peak at 1810.8644. NMR spectra of **4** and **1** were almost superimposable, the only difference being, as for compound **2**, the length of the hydrocarbon chain that has two extra methylene groups, confirmed by the presence, in the ESI^−^-MS/MS spectrum, of the fragment at *m*/*z* 652.5 [aglycone − H]^−^ (C_38_H_70_O_7_N).

In conclusion, the bioassay guided fractionation of the dichloromethane extract of the marine sponge *Erylus cf. deficiens* afforded a glycolipid fraction showing IDO inhibitory activity, from which were isolated four new polyketide glycosides structurally related to erylusamines reported in congener sponges [6,7,8]. The identification of the glycolipid content of sponges is important, not only due to the bioactivity that they usually display, but also because they have become useful markers in the taxonomic classification.

## 3. Materials and Methods

### 3.1. General Experimental Procedures

NMR spectra were acquired on a Bruker DRX-600 apparatus (Bruker BioSpin GmbH, Rheinstetten, Germany) operating at 600 for ^1^H and 150 MHz for ^13^C). Chemical shifts were expressed as δ values and reported to the residual solvent signals (pyridine-*d*_5_, δ_H_ = 8.73, 7.58 and 7.21; δ_C_ = 149.9, 135.5 and 123.5); coupling constants were reported in units of Hertz (Hz). HR-ESI-MS analysis was run on a Q-Exactive mass spectrometer (Thermo Fisher Scientific, Rockford, IL, USA). ESI-MS/MS spectra were achieved on a Q-Tof *micro* mass spectrometer (Waters, Milford, MA, USA). GC-MS analysis were performed on a Shimadzu GCMS-QP 2010Plus (Kyoto, Japan) using a Teknokroma TRB-1 column (30 m × 0.25 μm) (Barcelone, Spain).

IR spectra were obtained using a Mattson Satellite FT-IR (Waltham, MA, USA) and only the diagnostic absorption bands are reported, in cm^−1^. Flash column chromatography was performed on reversed-phase silica gel LiChroprep^®^ RP-18 40–63 μm (Merck Ref. 113900, Darmstadt, Germany). Thin layer chromatography was performed on silica gel 60 F_254_ aluminum sheets (Merck Ref. 5554) and visualized with UV light (254 nm) and vanillin/sulfuric reagent (0.5 g vanillin in sulfuric/MeOH 4:1 *v/v*) followed by heating up to 120 °C.

HPLC separations were performed on an Ultimate 3000 Dionex liquid chromatograph (Germering, Germany) equipped with a Phenomenex Luna 2.6 μ phenyl-hexyl column 100 Å (150 mm × 4.60 mm) (Torrance, LA, USA).

All solvents and reagents were obtained from commercial suppliers and were used without further purification.

### 3.2. Biological Material

A specimen of *Erylus cf*. *deficiens* Topsent, 1927 (Demospongiae, Tetractinellida, Geodiidae) was collected by scuba diving on the Gorringe Bank, a seamount located 150 km off the southwest coast of Portugal, at a depth between 40 and 50 m, and kept at −20 °C until processed. Identification was performed through analyses of the skeletal characters (spicules) under optical microscopy. A voucher sample was preserved in 90% ethanol and deposited in the Biology Department’s zoological collection of the University of the Azores, Ponta Delgada, Portugal (collection DBUA.Por).

### 3.3. Extraction and Isolation Procedures

The lyophilized specimens (63 g) were triturated in a grinder and extracted with methanol at room temperature for 24 h, yielding 7.2 g of crude extract after solvent evaporation under vacuum. This methanol extract was subsequently re-extracted with dichloromethane for 24 h, at room temperature, affording 1.8 g of extract. An aliquot of the dichlorometane extract (0.958 g) was coarse fractionated by RP-C18 flash chromatography with an eluent gradient of decreasing polarity from methanol to dichloromethane/methanol 9:1, in a total of 11 fractions. The more active fraction in the bioassay (fraction 2, 150 mg, eluent: methanol) was fractionated by HPLC using a column Phenomenex Luna 2.6 μ phenyl-hexyl 100 Å (150 mm × 4.60 mm) and a gradient of MeOH/0.1%TFA in H_2_O (flow 0.75 mL·min^−1^ from 80:20 to 100% MeOH). Erylusamides **A**–**D** (compounds **1**–**4**) were obtained by injection of more than two hundred 10 μL samples and pooling homologues fractions. Erylusamide **A**: (RT: 27.71 min, 27.7mg), Erylusamide **B**: (RT: 29.10 min; 23.0 mg), Erylusamide **C**: (RT: 30.74 min; 17.9 mg) and Erylusamide **D**: (RT: 33.41 min; 8.7 mg).

Erylusamide **A** (**1**): Colorless oil; ^1^H and ^13^C NMR data, see Table 2 and Table 3; HR-ESIMS *m*/*z* 1782.8345 [M − H]^−^ (calcd for C_83_H_132_O_40_N, 1782.8325); ESI-MS/MS *m*/*z* 828.5 [aglyconeGlc1 − H]^−^ (C_44_H_78_O_13_N), 624.5 [aglycone − H]^−^ (C_36_H_66_O_7_N).

Erylusamide **B** (**2**): Colorless oil; ^1^H and ^13^C NMR data, see Table 2 and Table 3; HR-ESIMS *m*/*z* 1796.8515 [M − H]^−^ (calcd for C_84_H_134_O_40_N, 1796.8482). ESI-MS/MS *m*/*z* 842.6 [aglyconeGlc1 − H]^−^ (C_45_H_80_O_13_N), 638.5 [aglycone − H]^−^ (C_37_H_68_O_7_N).

Erylusamide **C** (**3**): Colorless oil; ^1^H and ^13^C NMR data, see Table 2 and Table 3; HR-ESIMS *m*/*z* 1796.8434 [M − H]^−^ (calcd for C_84_H_134_O_40_N, 1796.8482). ESI-MS/MS *m*/*z* 842.6 [aglyconeGlc1 − H]^−^ (C_45_H_80_O_13_N), 638.5 [aglycone − H]^−^ (C_37_H_68_O_7_N).

Erylusamide **D** (**4**): Colorless oil; ^1^H and ^13^C NMR data, see Table 2 and Table 3; HR-ESIMS *m*/*z* 1810.8644 [M − H]^−^ (calcd for C_85_H_136_O_40_N, 1810.8638). ESI-MS/MS *m*/*z* 856.6 [aglyconeGlc1 − H]^−^ (C_46_H_82_O_13_N), 652.5 [aglycone − H]^−^ (C_38_H_70_O_7_N).

### 3.4. Methanolysis of Crude Fraction of Glycolipids

A portion of the crude fraction of glycolipids (12.9 mg) was dissolved in 1.5 mL of 2 M HCl in MeOH. The reaction mixture was stirred at 80 °C with refluxing for 4.5 h and, after cooling, neutralized with 5% ammonium hydroxide aqueous solution and finally evaporated to dryness under vacuum. The residue was partitioned between H_2_O and dichloromethane (2 mL × 3). Both phases were evaporated. The aglycone went into the organic phase and the methyl glycosides into the aqueous one.

Aglycone methyl ester (compound **5**, major/*minor rotamer):

^1^H NMR (pyridine-*d*_5_, 600 MHz): *δ* 5.38/4.93* (1H, q, *J* = 7.3Hz, H-2′); 3.96 (1H, m, H-26); 3.97 (1H, m, H-25); 3.63/3.67* (3H, s, CH_3_O); 2.94/2.97* (3H, s, H-3′); 2.40 (4H, m, H-15/H-17); 2.38 (2H, m, H-2); 1.88 (2H, m, H-23); 1.87 (2H, m, H-27); 1.77 (2H, m, H-3);1.60 (4H, m, H-14/H-18); 1.62 (2H, m, H-28); 1.41/1.46* (3H, d, *J* = 7.3Hz, H-4′); 1.32 (2H, m, H-29); 1.36 (2H, m, H-4); 1.28 (2H, m, H-13); 1.26 (24H, m); 0.85 (3H, t, *J* = 7.1Hz, H-30). ^13^C NMR (pyridine-*d*_5_, 150 MHz): *δ* 210.5 (C, C-16); 173.1/172.9* (C, C-1); 172.8/172.3* (C, C-1′); 75.2 (CH, C-26); 75.0 (CH, C-25); 53.2/55.6* (CH, C-2′); 51.3/52.2* (CH_3_, CH_3_O); 43.0 (CH_2_, C-15/C-17); 34.0 (CH_2_, C-2); 33.8 (CH_2_, C-24/C-27); 32.6 (CH_2_, C-28); 32.1/29.0* (CH_3_, C-3′); 29.6 (CH_2_, C-4/C-13); 27.1 (CH_2_, C-23); 25.4 (CH_2_, C-3); 24.3 (CH_2_, C-14/C-18); 23.1 (CH_2_, C-29); 15.0/15.9* (CH_3_, C-4′); 14.6 (CH_3_, C-30). HR-ESIMS *m*/*z* 620.4856 [M + Na]^+^ (calcd. for C_35_H_67_O_6_NNa, 620.4866). ESI-MS/MS *m*/*z* 620.5 [M + Na]^+^, 560.6, 503.5, 390.3, 265.3, 138.1.

### 3.5. Derivatization of Glycosides

The methyl glycosides were dissolved in 0.5 mL of pyridine and 36 μL of trimethylsilyl chloride (TMSCl) and 106 μL of hexamethyldisiloxane (HMDS) were added to the mixture. The reaction mixture was stirred at 60 °C for 2 h and evaporated to dryness. The residue was partitioned between H_2_O and dichloromethane (3 × 1 mL). The TMS-glycosides went into the organic phase and evaporated to dryness.

### 3.6. Preparation of Monosaccharide Standards

Commercial d-glucose, d-galactose and d-xylose were dissolved in 2 M HCl in MeOH and stirred with refluxing at 80 °C for 2 h. Thereafter, methanol and HCI were removed under a nitrogen stream without prior neutralization. An excess of TMSCl and HMDS were added to the dried material. The solutions were then heated at 60 °C for 2 h. The derivatized samples were evaporated under vacuum and used as standards for GC analysis

### 3.7. Synthesis of the Acetonide of Compound **5**

Compound **5** (0.77 mg, 1.2 μmol) was dissolved in dimethoxypropane (500 μL) with a catalytic amount of pyridinium *p*-toluenesulfonate (PPTS). The reaction mixture was heated at 60 °C for 5 h, then allowed to cool at room temperature and partitioned between water and Et_2_O (4 × 5 mL). The organic phase was evaporated to dryness under nitrogen stream affording compound **6** (0.8 mg, 1.2 μmol).

Acetonide of compound **5**:

^1^H NMR (pyridine-*d*_5_, 600 MHz): *δ* 5.37 (1H, q, *J* = 7.3Hz, H-2′); 3.74 (1H, m, H-26*); 3.72 (1H, m, H-25*); 3.62 (3H, s, OCH_3_); 2.94 (3H, s, H-3′); 2.43 (4H, m, H-15/H-17); 2.38 (2H, m, H-2); 1.65 (4H, m, H-14,/H-18); 1.51 (6H, s, acetonide α and β CH_3_); 1.41 (3H, d, *J* = 7.3Hz, H-4′); 1.36 (2H, m, H-4); 1.30 (2H, m, H-29); 1.28 (2H, m, H-13); 0.88 (3H, t, *J* = 7.3Hz, H-30). ^13^C NMR (pyridine-*d*_5_, 150 MHz): *δ* 210.6 (C, C-16); (173.2 (C, C-1); 172.7 (C, C-1′); 107.8 (C, acetonide OCO); 81.5 (CH, C-25*); 81.4 (CH, C-26*); 53.0 (CH, C-2′); 51.9 (OCH_3_); 42.7 (CH_2_, C-15/C-17); 33.3 (CH_2_, C-27); 31.9 (CH_3_, C-1′); 29.6 (CH_2_, C-4); 29.5 (CH_2_, C-13); 28.8 (CH_2_, C-28); 27.7 (acetonide CH_3_); 25.4 (CH_2_, C-3); 24.2 (CH_2_, C-14/ C-18); 23.0 (CH_2_, C-29); 14.6 (CH_3_, C4′) 14.2 (CH_3_, C-30).

### 3.8. Bioassay Description (GPSD^2^ Screening Application) [15]

Modified yeast cells from overnight growth are re-inoculated at OD 0.1 in selective medium to induce specific toxicity conditions and are dispensed automatically by a JANUS^®^ Automated Workstation (Perkin Elmer, Waltham, MA, USA) into a 96-well plate at a final volume of 200 μL.

In addition, 4 μL of organic and aqueous extracts (resuspended in dimethyl sulfoxide at a final concentration of 25 mg dry extract/mL) are added to 200 μL yeast cells, previously dispensed. One well is not exposed to any extract as control. Plates are incubated for 3 days. Absorbance and fluorescence signal were measured constantly every 2.5 h.

### 3.9. COS-7 Cells Bioassay [15]

COS-7 cells were grown in Dulbecco’s modified eagle medium (DMEM) 1000 mg/mL glucose, with GlutaMAX and pyruvate (Invitrogen, Carlsbad, CA, USA), supplemented with 10% fetal bovine serum (FBS) and 1% non-essential amino acids (NEAA). Cells were maintained at 80%–90% confluence at 37 °C and 5% CO_2_. COS-7 cells in 24-well plates were transiently transfected with pCDNA3-IDO using FuGene HD (Roche Diagnostics, Basel, Switzerland) following manufacturer’s instructions. In addition, 3 h post-transfection, 5 μL of samples’ stock solutions and 0.1× diluted solutions were added to cells and incubated for 24 h. Transfection efficiency after 24 h of extract exposure was assessed by direct observation of enhanced green fluorescent protein (EGFP) signal, using an inverted Carl Zeiss microscope AxioObserver D1 (Exc = 485/20 nm, Em = 515 nm) (Oberkochen, Germany). The IDO activity was evaluated by measuring kynurenine concentration in the supernatant by HPLC. Briefly, supernatants from cell culture were collected and immediately frozen at −20 °C until analysis. Protein precipitation and kynurenine extraction was performed by addition of trichloroacetic acid (TCA) at a final concentration of 6%. After discarding cell debris by centrifugation, supernatants were injected into the HPLC pump (Model LC-6A, Shimadzu Corporation, Kyoto, Japan). Separation was performed using a reversed-phase cartridge Aquasil RP18 column (200 mm length, 4.6 μm grain size) from Thermo Scientific (Rockford, IL, USA). An SPD-6AU UV-VIS spectrophotometric detector (Shimadzu Corporation, Kyoto, Japan) in a flow stream series connection was used for detection of kynurenine at a wavelength of 360 nm. The elution buffer consisted of a degassed potassium phosphate solution (0.015 mol/L, pH 6.4) containing 27 mL/L acetonitrile. Analysis was carried out at room temperature at a flow rate of 1.2 mL/min.

## Figures and Tables

**Table 1:** Glycolipid and lipid content of *Erylus* and *Pachymatisma* species.

Sponge/Origin	Compounds					
	Activity					
*Erylus formosus* La Parguera, Puerto Rico [4]	Fatty acid: Tetradecanoic 13-Methyltetradecanoic 12-Methyltetradecanoic 3-Methylpentadecanoic Hexadecenoic Methylpentadecanoic Hexadecanoic 3-Methylhexadecanoic 15-Methylhexadecanoic 14-Methylhexadecanoic 5,9-Octadecadienoic Octadecenoic Octadecanoic				Methyloctadecanoic 5,9-Icosadienoic 19-Methyl-5,9-icosadienoic 18-Methyl-5,9-icosadienoic Heneicosanoic Tricosanoic Tetracosanoic Pentacosanoic 24-Methyl-5,9-pentacosadienoic 5,9-Hexacosadienoic 25-Methyl-5,9-hexacosadienoic 24-Methyl-5,9-hexacosadienoic 5,9-Octacosadienoic 5,9-Nonacosadienoic	
	NR					
*Erylus goffrilleri* Mona Island (Puerto Rico) [5]	Fatty acid: Tridecanoic 12-Methyltridecanoic Tetradecanoic 3-Methyltetradecanoic 13-Methyltetradecanoic 12-Methyltetradecanoic 9-Pentadecenoic Pentadecanoic 3-Methylpentadecanoic 14-Methylpentadecanoic 13-Methylpentadecanoic (*Z*)-9-Hexadecenoic (*Z*)-11-Hexadecenoic Hexadecanoic (*Z*)-15-Methyl-9-hexadecenoic 10-Methylhexadecanoic 15-Methylhexadecanoic 14-Methylhexadecanoic (5*Z*,9*Z*)-2-Methoxy-5,9-hexadecadienoic (*Z*)-9-Heptadecenoic (*Z*)-11-Heptadecenoic Heptadecanoic (5*Z*,9*Z*)-2-Methoxy-15-methyl-5,9-hexadeca-dienoic Methylheptadecanoic (5*Z*,9*Z*)-5,9-Octadecadienoic (9*Z*)-2-Methoxy-15-methyl-9-hexadecenoic (*Z*)-9-Octadecenoic (*Z*)-11-Octadecenoic 2-Methoxy-14-methylhexadecanoic Octadecanoic Methyl-6-octadecenoic (5*Z*,9*Z*)-17-Methyl-5,9-octadecadienoic 11-Methyloctadecanoic (5*Z*,9*Z*)-2-Methoxy-5,9-octadecadienoic				(5*Z*,9*Z*)-2-Methoxy-5,9-nonadecadienoic 11-Eicosenoic Eicosanoic (5*Z*,9*Z*)-19-Methyl-5,9-eicosadienoic (5*Z*,9*Z*)-18-Methyl-5,9-eicosadienoic Methyleicosanoic (5*Z*,9*Z*)-5,9-Heneicosadienoic 19-Methyleicosanoic 18-Methyleicosanoic (5*Z*,9*Z*)-2-Methoxy-5,9-eicosadienoic 11-Nonadecenoic Nonadecanoic 5,8,11,14-Eicosatetraenoic Docosanoic 16-Methyldocosanoic 21-Methyldocosanoic 20-Methyldocosanoic Tricosanoic Methyltricosanoic Tetracosanoic Methyltetracosanoic (5*Z*,9*Z*)-24-Methyl-5,9-pentacosadienoic (5*Z*,9*Z*)-23-Methyl-5,9-pentacosadienoic (5*Z*,9*Z*)-5,9-Hexacosadienoic (5*Z*,9*Z*)-25-Methyl-5,9-hexacosadienoic (5*Z*,9*Z*)-24-Methyl-5,9-hexacosadienoic (5*Z*,9*Z*)-5,9-Heptacosadienoic (5*Z*,9*Z*)-26-Methyl-5,9-heptacosadienoic (5*Z*,9*Z*)-25-Methyl-5,9-heptacosadienoic (5*Z*,9*Z*)-5,9-Octacosadienoic (5*Z*,9*Z*)-5,9-Nonacosadienoic Methylnonadecanoic 17-Methyloctadecanoic 16-Methyloctadecanoic	
	NR					
*Erylus placenta* Hachijojima Island (Japan) [6,7]	Erylusamine A: R_1_ = CH_2_CH_2_CH_3_, R_2_ = H Erylusamine B: R_1_ = CH_2_CH(CH_3_)_2_, R_2_ = H Erylusamine C: R_1_ = CH_2_CH(CH_3_)_2_ R_2_ = Ac Erylusamine D: R_1_ = CH_2_CH_2_CH_2_CH_2_CH_3_ R_2_ = Ac			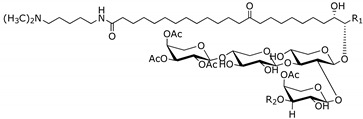		
	Interleukin-6 (IL-6) receptor antagonists					
*Erylus cf. Lendenfeidi* Gulf of Eilat (Red sea) [8]	Erylusamine TA: R_1_ = Ac; R_2_ = (CH_2_)_5_N(CH_3_)_2_; R_3_ = H, *n* = 8, *m* = 2 Erylusine: R_1_ = Ac; R_2_ = (CH_2_)_3_NCH_3_(CH_2_)_4_N(CH_3_)_2_; R_3_ = H, *n* = 8, *m* = 2 Erylusidine R_1_ = H; R_2_ = (CH_2_)_4_NHC = NH(NH_2_); R_3_ = COCH_2_CH(CH_3_)_2_, *n* = 8, *m* = 3					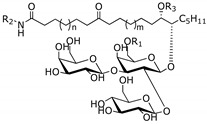
	NR					
*Erylus trisphaerus* Dominica [9]	Trisphaerolide	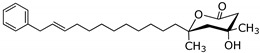				
	Low in vitro cytotoxicity against MCF7 human breast cancer cells					
*Pachymatisma johnstonia* Isle of Mann (UK) [3]	Pachymoside A		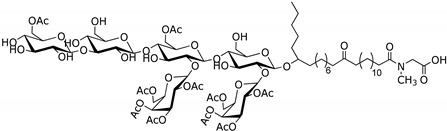			
	Crude extract showed inhibitory activity of bacterial type III secretion					

NR: not reported.

**Table 2:** NMR data for the aglycone **^a^** moieties of erylusamides **A**–**D** (**1**–**4**) in pyridine-*d*_5_.

1			2			3			4		
N°	δ ^13^C	δ ^1^H, m (*J*, Hz)	N°	δ ^13^C	δ ^1^H, m (*J*, Hz)	N°	δ ^13^C	δ ^1^H, m (*J*, Hz)	N°	δ ^13^C	δ ^1^H, m (*J*, Hz)
**1**	173.2	-	**1**	173.2	-	**1**	173.2	-	**1**	173.2	-
	173.1	-		173.1	-		173.1	-		173.1	-
**2**	33.8	2.43, m	**2**	33.8	2.43, m	**2**	33.8	2.43, m	**2**	33.8	2.43, m
**3**	25.5	1.81, m	**3**	25.5	1.81, m	**3**	25.5	1.81, m	**3**	25.5	1.82, m
**4**	29.7	1.38, m	**4**	29.8	1.38, m	**4**	29.8	1.39, m	**4**	29.7	1.38, m
**5–12**	29.6–29.9	1.18–1.33 Overlap.	**5–12**	29.6–29.9	1.18–1.33 Overlap.	**5–12**	29.7–29.9	1.18–1.32 Overlap.	**5–12**	29.6–29.9	1.19–1.33 Overlap.
**13**	29.6	1.28, m	**13**	29.6	1.28, m	**13**	29.6	1.28, m	**13**	29.6	1.28, m
**14**	24.2	1.64, m	**14**	24.2	1.64, m	**14**	24.2	1.64, m	**14**	24.2	1.65, m
**15**	42.8	2.42, m	**15**	42.7	2.42, m	**15**	42.8	2.42, m	**15**	42.8	2.42, m
**16**	210.5	-	**16**	210.5	-	**16**	210.5	-	**16**	210.5	-
**17**	42.8	2.42, m	**17**	42.7	2.42, m	**17**	42.8	2.42, m	**17**	42.8	2.42, m
**18**	24.2	1.64, m	**18**	24.2	1.64, m	**18**	24.2	1.64, m	**18**	24.2	1.65, m
**19–23**	29.6–29.9	1.19–132 Overlap	**19–24**	29.6–29.9	1.18–1.33 Overlap	**19–24**	29.7–29.9	1.18–1.32 Overlap	**19–25**	29.7–29.9	1.19–1.33 Overlap
**24**	29.7	1.83, m	**25**	29.7	1.82, m	**25**	29.7	1.81, m	**26**	29.7	1.83, m
**25**	74.9	5.53, m	**26**	74.9	5.53, m	**26**	74.9	5.54, m	**27**	74.9	5.54, m
**AcO**	169.7 *		**AcO**	169.7 *		**AcO**	169.7*		**AcO**	169.7 *	
**26**	80.7	4.03, m	**27**	80.8	4.03, m	**27**	80.7	4.03, m	**28**	80.8	4.05, m
**27**	30.9	1.79, m	**28**	30.9	1.79, m	**28**	39.1	1.16, m	**29**	30.9	1.80, m
**28**	32.0	1.23, m	**29**	32.0	1.23, m	**29**	28.2	1.51, m	**30**	32.0	1.25, m
**29**	22.8	1.28, m	**30**	22.8	1.28, m	**30**	22.8	0.85, d (6.0)	**31**	22.9	1.27, m
**30**	14.7	0.84, t (7.0)	**31**	14.2	0.86, t (6.5)	**31**	22.8	0.85, d (6.0)	**32**	14.2	0.86, t (6.6)
						**32**	22.8	0.85, d (6.0)			
**1’**	174.9	-	**1’**	174.9	-	**1’**	174.9	-	**1’**	175.0	-
	174.5	-		174.5	-		174.5	-		174.6	-
**2’**	52.7	5.75, q (7.2)	**2’**	52.6	5.74, q (7.3)	**2’**	52.7	5.74, q (7.3)	**2’**	52.7	5.75, q (7.3)
	55.8	4.97, q (7.3)			4.97, q (7.4)		55.7	4.97, q (7.3)		55.8	4.97, q (7.2)
**3’**	31.5	3.06, s	**3’**	31.5	3.06, s	**3’**	31.5	3.06, s	**3’**	31.5	3.07, s
	28.9	3.14, s		28.9	3.13, s		28.9	3.14,s		28.9	3.15,s
**4’**	15.0	1.54, d (7.3)	**4’**	15.0	1.55, d (7.3)	**4’**	15.0	1.55, d (7.3)	**4’**	15.0	1.54, d (7.4)
	16.0	1.60, d (7.2)		16.0	1.69, d (7.1)		16.0	1.60, d (7.3)		16.0	1.61, d (7.2)

^a^ duplicated values correspond to the major *syn* and minor *anti* rotamers respectively; * Overlapped with C-4 Glc3.

**Table 3:** NMR data for the carbohydrate moieties of erylusamides **A**–**D** (**1**–**4**) in pyridine-*d*_5_.

Position	1		2		3		4	
	δ ^13^C	δ ^1^H, m (*J*, Hz)	δ ^13^C	δ ^1^H, m (*J*, Hz)	δ ^13^C	δ ^1^H, m (*J*, Hz)	δ ^13^C	δ ^1^H, m (*J*, Hz)
**Gal**								
**1**	104.4	5.16, d (7.5)	104.4	5.16, d (7.7)	104.4	5.15, d (7.6)	104.4	5.16, d (7.7)
**2**	70.7	4.54, m	70.7	4.53, m	70.7	4.53, m	70.72	4.53, m
**3**	84.4	4.24, m	84.3	4.25, m	84.3	4.25, m	84.32	4.24, m
**4**	69.0	4.70, brs	68.9	4.71, brs	68.7	4.71, brs	68.91	4.72, brs
**5**	77.3	4.21, m	77.4	4.20, m	77.3	4.21, m	77.27	4.20, m
**6**	61.6	4.38, dd	61.6	4.38, dd	61.6	4.38, dd	61.62	4.38, dd
		(5.1; 10.6)		(5.2; 10.6)		(4.9; 10.9)		(5.2; 10.6)
		4.44, dd		4.43, dd		4.44, dd		4.44, dd
		(6.8; 10.6)		(6.9; 10.6)		(6.7; 10.9)		(6.9; 10.6)
**Xyl**								
**1**	103.2	4.90,d (7.3)	103.2	4.89, d (7.3)	103.2	4.90, d (7.6)	103.2	4.90, d (7.4)
**2**	78.3	4.22, m	78.2	4.22, m	78.3	4.21, m	78.3	4.21, m
**3**	88.2	4.25, m	88.1	4.24, m	88.0	4.24, m	88.0	4.26, m
**4**	69.0	4.00, m	68.9	3.99, m	69.0	3.99, m	69.0	4.00, m
**5**	65.9	3.51, t (10.7)	65.9	3.50, t (9.8)	65.9	3.51, t (10.3)	65.9	3.51, t (10.7)
		4.14, m		4.14, m		4.14, m		4.13, m
**Glc1**								
**1**	104.6	4.86, d (7.9)	104.6	4.87, d (7.8)	104.6	4.88, d (8.0)	104.6	4.87, d (8.0)
**2**	74.9	3.97, m	74.6	3.96, m	74.6	3.97, m	74.6	3.98, m
**3**	76.6	4.18, m	76.6	4.18, m	76.6	4.18, m	76.6	4.18, m
**4**	81.9	3.97, m	81.8	3.97, m	81.9	3.97, m	81.9	3.97, m
**5**	73.0	4.09, m	72.9	4.08, m	73.0	4.10, m	73.0	4.11, m
**6**	64.3	4.92, m	64.3	4.93, m	64.1	4.93, m	64.3	4.93, m
		5.16, m		5.14, m		5.16, m		5.15, m
**Ac (C-6)**	171.0	-		-	171.0		171.0	-
**Glc2**								
**1**	102.8	5.55, d (8.0)	102.8	5.55, d (8.2)	102.8	5.55, d (8.5)	102.8	5.55, d (8.1)
**2**	72.2	5.48, dd	72.2	5.47,dd	72.2	5.48, t	72.1	5.48, dd
		(8.2; 9.5)		(8.4; 9.3)		(9.3)		(8.4;9.3)
**3**	73.5	5.78, t (9.6)	73.5	5.78, t (9.6)	73.5	5.78, t (9.9)	73.4	5.78, t (9.5)
**4**	69.4	5.42, t (9.8)	69.4	5.42, t (9.7)	69.4	5.42, t (9.7)	69.4	5.42, t (9.7)
**5**	72.1	4.22, m	72.1	4.21, m	72.1	4.22, m	72.1	4.22, m
**6**	62.7	4.32, dd	62.7	4.32, dd	62.6	4.31, dd	62.7	4.31, dd
		(2.3; 12.1)		(2.1; 11.8)		(~2; 11.8)		(2.3;12.1)
		4.51, dd		4.50,dd,		4.52, dd		4.51, dd
		(5.4; 12.1)		(5.3; 12.0)		(4.8; 11.8)		(5.4;12.1)
**Ac (C-2)**	170.2	-	170.2	-	170.2	-	170.2	-
**Ac (C-3)**	170.1	-	170.1	-	172.2	-	170.2	-
**Ac (C4)**	169.8	-	169.9	-	169.9	-	169.9	-
**Ac (C-6)**	170.4	-	170.5	-	170.5	-	170.5	-
**Glc3**								
**1**	99.6	6.37, d (7.9)	99.6	6.35, d (8.0)	99.6	6.36, d (7.6)	99.6	6.36, d (7.9)
**2**	72.3	5.62, m	72.2	5.61, t (10)	72.2	5.62, m	72.2	5.62, m
**3**	73.7	5.90, t (9.4)	73.7	5.89, t (9.5)	73.7	5.9, t (9.3)	73.7	5.90, t (9.5)
**4**	69.7	5.58, m	69.7	5.56, m	69.7	5.56, m	69.7	5.56, m
**5**	72.3	3.93, m	72.2	3.92, m			72.2	3.92, m
**6**	62.5	4.19, m	62.5	4.56, m	62.4	4.19, m	62.4	4.18, m
		4.57, m		4.18, m		4.56, m		4.57, m
**Ac(C-2)**	169.6	-	169.6	-	169.7	-	169.7	-
**Ac(C-3)**	170.5	-	170.5	-	170.5	-	170.5	-
**Ac(C-4)**	169.7	-	169.7	-	169.8	-	169.7	-
**Ac(C-6)**	170.2	-	170.4	-	170.4	-	170.4	-
